# *In vitro* Engineering of Novel Bioactivity in the Natural Enzymes

**DOI:** 10.3389/fchem.2016.00039

**Published:** 2016-10-07

**Authors:** Vishvanath Tiwari

**Affiliations:** Department of Biochemistry, Central University of RajasthanAjmer, India

**Keywords:** *in vitro* design, strategic manipulation, molecular evolution, novel bioactivity of natural enzyme, thermodynamics of *in vitro* design

## Abstract

Enzymes catalyze various biochemical functions with high efficiency and specificity. *In vitro* design of the enzyme leads to novel bioactivity in this natural biomolecule that give answers of some vital questions like crucial residues in binding with substrate, molecular evolution, cofactor specificity etc. Enzyme engineering technology involves directed evolution, rational designing, semi-rational designing, and structure-based designing using chemical modifications. Similarly, combined computational and *in vitro* evolution approaches together help in artificial designing of novel bioactivity in the natural enzyme. DNA shuffling, error prone PCR and staggered extension process are used to artificially redesign active site of enzyme, which can alter its efficiency and specificity. Modifications of the enzyme can lead to the discovery of new path of molecular evolution, designing of efficient enzymes, locating active sites and crucial residues, shift in substrate, and cofactor specificity. The methods and thermodynamics of *in vitro* designing of the enzyme are also discussed. Similarly, engineered thermophilic and psychrophilic enzymes attain substrate specificity and activity of mesophilic enzymes that may also be beneficial for industry and therapeutics.

## Introduction

Enzymes are the biocatalysts that have specificity, efficiency, and mode of regulation. They are also used for varied industrial processes especially in medical research such as *de novo* drug discovery and its improvement (increase the yield of antibiotics, hormones, etc.), crop improvement, biofuel industry, food industry, etc. Enzymes used in biotechnological process have to be very efficient in their catalytic activity because *in vivo* and *in vitro* conditions are very different. Most of the enzymes are derived from mesophilic organisms; hence they are most active in moderate conditions. Therefore, in order to improve the catalytic performance of the industrial enzymes and withstand harsh conditions, protein engineering plays a major role, and resulting in the reduction of cost and time of processing in the respective industries. Changing cofactor specificity, discovering path of molecular evolution, designing more powerful and efficient enzymes, locating active sites and crucial residues, shifting the substrate specificity toward new substrate, improving binding affinity, changing in pH tolerance and inhibitor tolerance, improving solubility and resistance to extreme conditions, are some of the modifications that can be achieved by *in vitro* enzyme designing. Enzymes can be altered by redesigning the active site that can alter its efficiency (k_cat_/k_m_) and specificity (k_m_). Altering the ligand binding sites, incorporation of new functional groups into the active site, DNA shuffling, site-saturation, and site-directed mutagenesis are some of the approaches that modify the active site of an enzyme. Thermo-stabilizing traits such as high number of hydrogen bonds, introduction of ion pairs for linking of N and C-terminals, addition of proline residues in loops are among many modifications that can be achieved at genetic level through mutagenesis after complete analysis of sequence and structure of homologous mesophilic and thermophilic enzymes. Cryo-stabilizing characteristics such as clustering of glycine residues which provide local flexibility in the region, reduced frequency of proline residues in loops leading to enhanced chain flexibility between secondary structures, a reduction in arginine residues which generally participate in formation of multiple salt bridges and H-bonds, and a lowered number of ion pairs and aromatic interactions, can also be introduced by mutagenesis. These characteristics of thermophilic and cryophilic enzyme can be added in the mesophilic enzyme.

Theodosius Dobzhansky stated that “Nothing in biology makes sense except in the light of evolution” (Dobzhansky, 1973). Evolution is the change in heritable phenotypic trait of biological population over successive generations. The amazing similarity in gene sequences and metabolic pathways across the phyla says that modern organisms are derived from common progenitor by series of small changes, i.e., mutations. Homology means two proteins are descended from a common ancestor while sequence homology means amino acids at equivalent positions are similar because they are descended from a common ancestor (Hall and Barlow, 2004). The gradual accumulation of mutations over long period of time results in the formation of new species. Natural *in vivo* design of the bioactivity leads to the molecular evolution. Molecular evolution is a fundamental biological process and its understanding would facilitate the engineering of enzymes with new specificities. During the course of evolution, it is possible that inefficient enzymes convert to efficient enzymes or vice-versa (Geddie and Matsumura, 2004). The *in vitro* design of the activity can be done using various mutagenesis approaches. Thus, enzyme engineering using *in vitro* approaches may contribute to elucidate structural basis of the functional properties, and the course of the natural molecular evolution. In the present review, we have explained the methods for *In vitro* mutagenesis and their significances in the enzyme substrate replacement, cofactor replacement, and change in enzyme catalysis.

## Methods for *in vitro* design or artificial mutation

**Figure 1:**
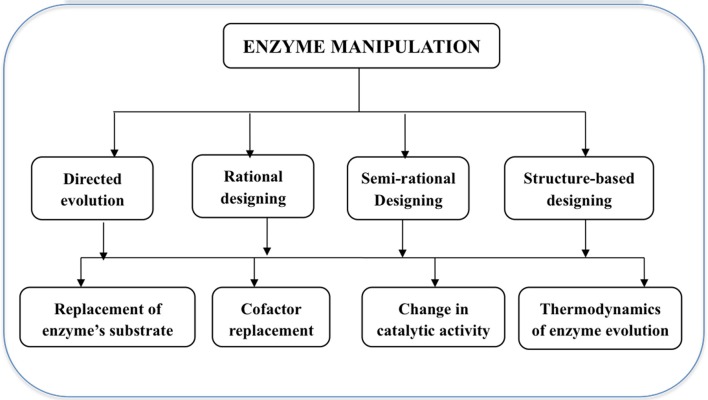
**Summary of enzyme engineering methods and their significances**.

Protein engineering technology (Figure 1) involves directed evolution, rational designing, semi-rational design, structure-based design using chemical modification, and truncated and fusion methods (Li and Cirino, 2014; Kaushik et al., 2016). In directed evolution, natural evolution is mimicked in the lab and based on developing molecular diversity by random mutagenesis and *in vitro* recombination followed by performing high-throughput screening for improvements in desired phenotype. Similarly, rational protein design is used for those enzymes whose three-dimensional structures have already been determined and site-directed mutagenesis is used to introduce specific and deliberate mutations on the enzyme sites. Both directed evolution and rational protein design have certain limitations, hence a combined approach known as semi-rational design, takes advantage of already available information of structure and function of the desired protein and designs a smaller but higher quality library. Molecular dynamics and QM studies also compliment such studies as they facilitate the understanding about the impact of every single amino acid on the structure and function of the protein. This computer-guided semi-rational designing of proteins further helps in shaping the needs of a good biocatalyst with a larger substrate range, selectivity, specificity, and stability without harming their catalytic efficiency (Kaushik et al., 2016).

Recent studies have shown that combined computational and *in vitro* evolution approaches can be used for the study of evolution of novel bioactivity in the natural enzymes. *In vitro* study can be achieved by construction of large libraries of protein/enzyme mutants, enzyme with certain substrate binding characteristics are selected, and the sequences of the selected enzyme are determined. The main advantage of this method is that they require minimal knowledge of enzyme substrate interaction under study. This method does not provide much information about the nature of intermolecular contacts, which often hampers an understanding of the obtained results. Computational protein design is parallel approach that is based on our understanding of the biophysical forces that govern enzyme-substrate binding but it require a detailed knowledge of the structure and function of the enzyme substrate under study (Rosenfeld et al., 2016).

Other approach using novel scaffolding and compartmentalization techniques are also used to improve performance of enzyme (Li and Cirino, 2014). Consensus design is another approach that can be used to artificial designing of enzyme, which is based on the hypothesis that at a given position of enzyme, the respective consensus amino acid contributes more than average to the stability of the enzyme than non-conserved amino acids. Consensus design, like ancestral sequence reconstruction, utilizes evolutionary history; all sequences are aligned, and most frequently observed amino acid is identified at each position in the alignment (Porebski and Buckle, 2016). To achieve the molecular evolution of a protein, a consensus library for directed evolution experiments could be made, which would contain information about site-directed mutations of a particular protein. This mimics the natural Darwininan evolution process, which involves repeated cycles of mutation, followed by selection that renders a strong algorithm to create diversity as exhibited by the plethora of life. The process can be performed *in vitro* by introducing random mutations on template strand, followed by screening and selection of desired mutant clones. The whole procedure is repeated until desired mutation is achieved. Selection or screening of a consensus library should reveal correlative or co-varying substitutions that change parallel in order to alter protein function (Flores and Ellington, 2005). Redesigning of enzymes does not only require change in the residues that bind to substrates and cofactor but also requires residues that are far from active site but important to maintain catalytic residues in prerequisite orientations. Electrostatic charge distribution throughout a whole enzyme molecule may also facilitate substrate binding by electrostatically guiding the substrate into the active site (Oue et al., 1999). The mutagenized gene library is made by various methods such as DNA shuffling, error prone PCR (ePCR) and staggered extension process (StEP). However, the efficiency rate of directed evolution is low and less yield of the final PCR product acts as a drawback for the downstream processing. This can be avoided using ExoSAP-IT PCR Product Cleanup (Affymetrix), that serves as an alternative to beads based PCR (Goh et al., 2012).

ePCR incorporates error in the final product during DNA amplification. Errors are introduced by altering the parameters of PCR such as use of high concentrations of magnesium or manganese, DNA polymerase not capable of proofreading, increase in the number of amplification cycles and/or biased concentration of dNTPs. DNA shuffling is one of the most important directed evolution techniques which has increased the catalytic efficiency of various industrial enzymes namely β- 1,3- 1,4-glucanase (Yan et al., 2006), glucose dehydogenase (Gruber et al., 1998) etc. This technique involves formation of various chimeras by joining different homologous genes via self-priming PCR and ultimately creating a library. More than 80% similarities should be there in homologous parent genes (Brakmann and Schwienhorst, 2004). StEP is also a commonly used homology-dependent process for performing *in vitro* DNA recombination. For the purpose of efficient recombination the parental genes must share more than 85% sequence identity (Volkov and Arnold, 2000).

Recently, mutagenesis methods have improved tremendously in comparison to earlier laborious primer extension methods that require templates (to be mutated) in single stranded form as well as stringent screening procedures for selection of mutants. Advance methods such as Exsite and GeneTailor uses double stranded template DNA and PCR to mutate the targets followed by easy selection of mutants. Alanine scan is a standard method to analyze the active site mechanism and identify the role of important residues, where the concerned residues are mutated with alanine (Dembowski and Kantrowitz, 1994). Hence with the types of technologies available today and with the widely accessible information (web based databases), engineering procedures have become routine and are continuously introducing new enzymes and proteins into market with enhanced traits or novel properties for therapeutic, pharmaceutical and industrial applications. *In vitro* mutagenesis results from the three type of changes i.e., enzyme substrate replacement, cofactor replacement, and change in enzyme catalysis. We have critically analyzes each of these in detail by taking examples of different enzymes. Various enzymes and their mutants formed by site-directed mutagenesis along with the mutation sites are listed in Table 1.

**Table 1:** **Enzymes and their mutants formed by site directed mutagenesis**.

**S. no**.	**Source**	**Wild type enzyme**	**Substrate specificity/cofactor specificity**	**Mutant enzyme**	**New substrate specificity/cofactor specificity**	**Mutations at specific sites**	**References**
1.	*Trichomonas vaginalis*	Lactate dehydrogenase	Lactate etc.	Malate dehydrogenase	Malate etc.	Leu91Arg	Wu et al., 1999
2.	*Citrobacter freundii*	Tyrosine phenol lyase	β- elimination of tyrosine	Dicarboxylic amino acid β-lyase enzyme	Reversible transfer of an amino group from dicarboxylic amino acids to oxo acids	Arg100Thr, Val283Arg	Mouratou et al., 1999
3.	*Citrobacter freundii*	Tyrosine phenol lyase	β- elimination of tyrosine	Tyrosine phenol lyase	Decreased affinity for β- elimination of tyrosine	Asn185Ala, Tyr71Phe, Thr124Asp, Phe448His	Chen H. Y. et al., 1995; Barbolina et al., 2000; Demidkina et al., 2002
4.	*Lactobacillus pentosus*	NAD-dependent D-lactate dehydrogenase	Pyruvate	D-2hydroxyisocaproate dehydrogenase	Larger aliphatic or aromatic 2-ketoacid substrates	Tyr52Leu	Tokuda et al., 2003
5.	*E. coli*	Choline acetyl transferase	Acetyl group acceptor is choline	Carnitine acetyl transferase	Specificity More for carnitine and less for choline	Val459Thr, Asp460Glu, Asn461Thr Asn514Arg	Reznik et al., 1998
6.	*E. coli*	Aspartate amino- transferase	reversible transfer of the amino group of aspartate or glutamate to the cognate oxo acids	L-aspartate-β decarboxylase	β-caboxylase activity	Tyr225Arg, Arg292Lys Arg386Ala	Graber et al., 1999
7.	*Bacillus stearothermophilus*	Lactate dehydrogenase	specific for cofactor NAD_+_	Same	Utilized NADP^+^ far better	Phe16Gln, Cys81Ser Asn85Arg	Flores and Ellington, 2005
8.	*E. coli*	β-Glucuronidase	β- galactosidase activity	Same	Increased in β galactosidase activity	Thr509Ala, Ser557Pro, Asn566Ser Lys568Gln	Geddie and Matsumura, 2004
9.	*E. coli*	Malate dehydrogenase	Inter-conversion of malate & oxaloacetate	Same	Catalytic activity is decreased & specificity for other substrate increased	Arg153Cys	Wright and Viola, 2001
10.	*E. coli*	Aspartate amino transferases	Aspartate	Same	Five-fold increase in activity	Asn34Asp, Ile37Met, Ser139Gly, Asn142Thr, Asn297Ser, Val387Leu	Yano et al., 1998
11.	*E. coli*	Aspartate amino transferases	Aspartate	same	Valine	17 mutations	Oue et al., 1999
12.	*E. coli*	Streptavidin	Biotin	Same	Biotin analogs e.g., Iminobiotin	Asn23Ala Ser27Asp	Reznik et al., 1998
13.	*Klebsiella pneumoniae*	1,2 propanediol oxidoreductase	Coenzyme-NADH	Same	Coenzyme-NADH and NADPH	Asp41Ala, Asp41Gly	Ma et al., 2010
14.	*Bacillus lentus*	Subtilisins	Proteolytic activity	Same	Increased proteolytic activity	Lys27Arg/ Asn87Ser/ Val104Tyr/ Asn123Ser /Thr274Ala Asn76Asp/ Asn87Ser/ Ser103Ala/ Val104Ile	Graycar et al., 1999
15.	*Streptomyces* spp.	Cholestrol oxidase	Pregnenolone, cholesterol	Same	Increased catalytic activity	Ser379Thr	Toyama et al., 2002
16.	*E. coli*	Isocitrate dehydrogenase	Coenzyme-NADP	Same	Coenzyme-NAD	6 mutations	Chen R. et al., 1995
17.	*Peptostreptococcus asaccharolyticus*	Glutamate dehydrogenase	Coenzyme-NADH	Same	Coenzyme-NADPH	Glu243Lys/Glu243Asp	Carrigan and Engel, 2007
18.	*Candida boidinii*	Formate dehydrogenase	Coenzyme-NAD^+^	Same	Coenzyme-NADP^+^	Asp195Gln/Tyr196His	Andreadeli et al., 2008
19.	*E. coli*	Malate dehydrogenase	Oxaloacetate	Phenylactate dehydrogenase	Phenyl pyruvate	Arg81Cys	Wright et al., 2000
20.	*Streptomyces coelicolor*	Malate dehydrogenase	NADP	same	NADPH	Glu42Gly, Ile43Ser, Pro45Arg, Ala46Ser	Ge et al., 2014
21.	*Pseudomonas* N176	Glutaryl-7-ACA acylase	CephC	CephalosporinCacylases (CA)	CephC	His57Ser His70Ser, Leu154Tyr	Conti et al., 2014
22.	*Candida tenuis*	Xylose reductase	NADPH	Same	NADH	Lys274Arg–Asn276Asp	Petschacher et al., 2005
23.	*Thermus thermophilus*	Lactate dehydrogenase	NADH	Same	NADPH	7 amino acid residues	Tomita et al., 2006b
24.	*Pichia stipitis*	Xylose reductase	NADPH	Same	NADH	Lys21Ala/ Asn272Asp	Zeng et al., 2009
25.	*Clostridium symbiosum*	Glutamate dehydrogenase	NADH	Same	NADPH	Asp263Lys, Phe238Ser, Pro262Ser	Griffin and Engel, 2011
26.	*Thermus flavus*	Malate dehydrogenase	NADH	Same	NADPH	7 amino acids residues	Nishiyama et al., 1993; Tomita et al., 2006a
27.	*E. coli*	Lactaldehyde dehydrogenase	NADH	Same	NADPH	Phe180Thr	Rodríguez-Zavala, 2008
28.	*Candida magnoliae*	Carbonyl reductase	NADPH	Same	NADH	8 amino acid residues	Morikawa et al., 2005
29.	*Neurospora crassa*	Nitrate reductase	NADPH	Same	NADH	Ser920Asp Arg932 Asp	Shiraishi et al., 1998
30.	*Lactobacillus bulgaricus*	Lactate dehydrogenase	NADH	Same	NADPH	Asp175Ala	Bernard et al., 1995
31.	*Glomerella cingulata*	Cutinase	Hydrolysis of esters and triglycerides	Same	Catalytically inactive	His204Asn	Nyon et al., 2009
32.	*Pseudomonas aeruginosa* LST- 03	Lipase	Ester synthesis and inter-esterification reaction and lipid hydrolysis	Same	Increased organic solvent stability	Ser155Leu Gly157Arg, Ser164Lys, Ser194Arg, Asp209Asn	Kawata and Ogino, 2010
33.	*Geobacillus kaustophilus* HTA426	Lactonase	3-oxo-N-dodecanoyl-L-homoserine lactone	Same	72-fold increase in the catalytic efficiency	Glu101Asn/ Arg230Ile	Chow et al., 2010
			3-oxo-N-dodecanoyl-L-homoserine lactone	Same	*N*-butyryl-l-homoserine lactone	Asp266Asn	Chow et al., 2010
34.	alkalophilic Bacillus sp.	Cyclodextrin glucano transferase	produce cyclodextrins from starch	Same	higher product yields	His233Tyr	Leemhuis et al., 2010
35.	*E. coli*	Lactaldehyde reductase	NADH	Same	3.6-fold increase in k_cat_	Met185Cys	Cahn et al., 2016
36.	*Saccharomyces cerevisae*	Cinnamyl alcohol dehydrogenase	NAD(P)H	Same	82-fold increase in activity	Gln110Val	Cahn et al., 2016
37.	*Actinoplanes utahensis*	Cephalosporin acyclase	Cephalosporin	Same	*Aculeacin A*	Phe177(β)Gly/Met145(α)Ala and Phe177(β)Gly/Met145(α)Ala/Tyr149(α)Val	Isogai and Nakayama, 2016

## Significance of *in vitro* manipulation

### Replacement of natural substrate with a novel substrate

The alteration in the substrate specificity is seen mainly in the dehydrogenases. The reason might be the utilization of similar cofactor i.e., NAD by all dehydrogenases. Malate dehydrogenase (MDH) from *Escherichia coli* is highly specific for its keto acid substrate. The replacement of one of the binding arginine residues (81st position) with unnatural aryl and alkyl amino acid analogs, result into enhancement of the specificity of MDH for phenyl pyruvate while diminished for oxaloacetate (physiological substrate; Wright et al., 2000). This is performed by site-specific modulation that incorporates systemic structural information at the target site. Similarly, some experiments also revealed that three arginines (80th, 87th, and 153th) of malate dehydrogenase are responsible for selectivity of the substrate. Site-directed mutagenesis at Arg153Cys was done which decreased its specificity for malate and oxaloacetate. This substitution changed the conformation of the active site and thus the specificity for original substrate decreased. Substitutions of functional group like acetamide etc. changed the substrate specificity to pyruvate, alpha-ketoglutarate etc. (Bell et al., 2001; Wright and Viola, 2001).

Some other studies reported that a single amino acid mutation (Tyr52Leu) was done in NAD-dependent D-lactate dehydrogenase of *Lactobacillus pentosus*, resulted in the increased affinity of enzyme toward larger aliphatic or aromatic 2-ketoacid substrates by four-fold as well as decreased affinity toward pyruvate (which is its actual substrate) by about 30-fold and thus converting the enzyme into D-2-hydroxyisocaproate dehydrogenase (an active enzyme; Tokuda et al., 2003).

Likewise, many attempts have been made to redesign the active sites of enzymes such as lactate dehydrogenase (LDH) present in *Trichomonas vaginalis (Tv)*. It resembles malate dehydrogenase (cytosolic) more closely than LDH of other species. The Arg at 91st position of MDH was replaced by Leu in *Tv*LDH. The change Leu91Arg was done by site directed mutagenesis that converted *Tv*LDH to MDH. The reverse change i.e., Arg91Leu does not yield a measurable activity of *Tv*LDH. The result was verified by molecular modeling and *in vitro* functional characterization. This conversion showed that MDH-LDH barrier has been broken in at least one direction. This also highlighted the role of some important residue for selection of catalysis of a substrate. Phylogenetic reconstructions showed that members of the MDH and LDH families form separate monophyletic lineages probably arose from early gene duplication (Wu et al., 1999). Similarly, studies suggest that evolution of (*Cryptosporidium parvum*) lactate dehydrogenase from malate dehydrogenase arose by gene duplication. Ancestral sequence reconstruction technique is useful to study these evolutionary paths (Madern et al., 2004).

Another enzyme, aspartate aminotransferases are the most extensively studied vitamin B-6 containing enzyme. These are homodimeric enzymes that catalyze amino group transfer between acidic amino acids, aspartate and glutamate, and their corresponding 2-oxo acids. The triple mutation at its substrate-binding site leads to increased rate of L-aspartate β-decarboxylase activity to transaminase activity by 25 million-fold. In order to elucidate both the structural basis of the functional properties and course of the molecular evolution, molecular dynamics, and crystal studies are performed (Graber et al., 1999). Similarly, directed molecular evolution was done by mutating 17 amino acids residues of enzyme aspartate aminotransferases in *E. coli*. This had resulted in six-fold increase in activity for non-native substrate i.e., valine. The remodeled enzyme had altered subunit interface, and a shift in the enzyme domain. These results clearly demonstrate the importance of the cumulative effects of residues remote from the active site and present a new line of approach to redesign enzymes (Oue et al., 1999).

Molecular dynamics and homology data also identified the critical residues of tyrosine phenol lyase (TPL), an enzyme involved in the β-elimination of tyrosine. Double mutations at R100T and V283R are responsible for switching the substrate specificity of TPL from L-tyrosine to dicarboxylic amino acids in *Citrobacter freundii*. These mutations increased its β-elimination activity toward dicarboxylic acids by four-fold without destroying its catalytic apparatus. Thus, this experiment gave a novel enzyme dicarboxylic amino acid β-lyase, which is not found in nature. From this study, it can be concluded that these enzymes originated from region-specific catalysts, which first specialized for reaction specificity and then for substrate specificity (Mouratou et al., 1999).

Site directed mutagenesis has been routinely used to redesign enzymes, but the achievement in this technique relies basically on the availability of 3-D structure protein maps. Others techniques that are based on cross-referencing substrate structure and comparisons with protein alignment data, were also used. Choline acetyltransferases reversibly transfer acetyl group between acetyl-coA and choline. After site-directed mutagenesis the mutant enzyme have more affinity for carnitine rather than choline. Thus, novel enzyme called carnitine/choline acetyltransferase was obtained (Cronin, 1998). Likewise, catalytic efficiency of cholesterol oxidase from *Streptomyces* and *Brevibacterium*, was also studied by site directed mutagenesis and found that efficiency was increased in the mutant S379T toward cholesterol and pregnenolone. These findings provide ideas for designing more efficient enzyme which can be clinically useful for cholesterol estimation (Toyama et al., 2002).

A well-known industrial biocatalyst Glutaryl acylase (GA), belongs to the N-terminal hydrolase class of hydrolytic enzymes and show wide substrate specificity. CA (cephalosporin C acylase) and GAs are members of the glutaryl acylase family that specifically use CephC as their substrate. GA undergoes a two-step reaction to yield cephalosporin and CA undergoes only one step reaction to yield cephalosporin. CAs isolated from *Pseudomonas* strains has very low activity for biotechnological use. By using protein engineering approach based on combined use of error-prone PCR mutagenesis, molecular modeling, site saturation and site-directed mutagenesis, the glutaryl acylase from *Pseudomonas* N176 has been mutated (A215αY-H57βS-H70βS and H57βS-H70βS-L154βY). These enzyme variants have 100-fold increase in specificity constant for CephC (Conti et al., 2014).

In addition to the alteration in the enzymes action, artificial mutation can also alter the proteins for non-catalytic function. Streptavidin and biotin is the model system to study structure and thermodynamic properties of high affinity receptor complexes with its ligand. Biotin binding sites were redesigned in such a way that the major structural motifs of the natural streptavidin-biotin complex are preserved. This was done by changing amino acid residues deep in the biotin-binding pocket to reduce the biotin-binding affinity considerably without disturbing the binding affinities for biotin analogs. Thus, a mutant of streptavidin was made which has greater binding specificity to 2-iminobiotin than to its natural ligand biotin. This approach could also be useful for *in vivo* targeting applications (Reznik et al., 1998).

### Alteration in coenzyme specificity of enzyme

Cofactor specificity can also be altered by using techniques like site-directed mutagenesis, DNA shuffling etc. The enzyme lactate dehydrogenase has a specific cofactor NAD^+^. Using DNA shuffling method, many mutants were made by substitution at cofactor binding sites. A consensus library was made to study cofactor specificity of *Bacillus stearothermophilus* lactate dehydrogenase. A triple mutant was selected which had decreased specificity for NAD^+^ and increased for NADP^+^. Thus, an enzyme with change in cofactor was made without decrease in its catalytic activity (Flores and Ellington, 2005). Similarly, the NAD-dependent D-lactate dehydrogenase from *Lactobacillus bulgaricus* had also been modified to display 40-fold shift of coenzyme specificity from NADH to NADPH (Bernard et al., 1995).

Similar experiments were also done in different organisms to change the cofactor specificity. Malate dehydrogenase (MDH) of *Streptomyces coelicolor* (*Sc*MDH) was altered by site-directed mutations in the Rossman fold region to change its cofactor specificity from NADH to NADPH. The coenzyme specificity (*K*_cat_/*K*_m_) of the mutant enzyme was examined and found to be shifted 2231.3-fold toward NADPH (Ge et al., 2014). Similarly, in *Thermus thermophilus, Thermus flavus*, and *Thermococcus kodakarensis* replacement of specific amino acids in Rossman fold of NADH dependent lactate dehydrogenase changed its cofactor from NADH to NADPH (Nishiyama et al., 1993; Tomita et al., 2006a; Morimoto et al., 2014).

Xylose reductase (NADPH) and xylitol dehydrogenase (NADH) of *Pichia stipiti* are involved in the catabolism of xylose and for ethanol production from it. As the cofactors of both these enzymes are different, redox imbalance occurs. To solve this problem, site directed mutagenesis was done at Lys21 in xylose reductase. Both K21A and K21A/N272D mutants preferred NADH over NADPH. Thus, a complete reversal of coenzyme specificity toward NADH and improved catalytic efficiency were achieved for this enzyme (Zeng et al., 2009). In *Candida tenuis*, similar experiments (single mutant K274R, K274M, K274G, S275A, N276D, R280H, and double mutant K274R-N276D) were done to change the cofactor specificity of xylose reductase (Petschacher et al., 2005).

Likewise, some residues of glutamate dehydrogenase of *Peptostreptococcus asaccharolyticus* and *Clostridium symbiosum* has been mutated, due to which catalytic efficiency toward NADPH and NADP^+^ was increased by several folds, respectively (Griffin and Engel, 2011). Formate dehydrogenase from *Candida boidinii* (CboFDH) is known to catalyse the oxidation of formate anion to carbon dioxide with concomitant reduction of NAD^+^ to NADH. It is highly specific to NADH and virtually fails to catalyze the reaction with NADPH, but substitution at Asp195Gln/Tyr196His position causes shift of cofactor specificity from NADH to NADPH (Andreadeli et al., 2008). In isocitrate dehydrogenase from *E. coli*, site-directed mutagenesis was used to introduce five substitutions (Lys344Asp, Tyr345Ile, Val351Ala, Try391Lys, Arg395Ser) in its adenosine binding pocket that shifts the coenzyme specificity from NADP to NAD (Chen R. et al., 1995). Studies also suggest that a mutation in phenylacetaldehyde dehydrogenase mutation at F180T in lactaldehyde dehydrogenase increases its specificity to NADP^+^ that also increased enzyme's catalytic activity (Rodríguez-Zavala, 2008).

Same approach was applied to mutate various other enzymes like nitrate reductase, carbonyl reductase etc. which have been highlighted in Table 1(Shiraishi et al., 1998; Morikawa et al., 2005). Microbial production of 1, 3-propanediol has been considered as a competitor to the traditional petrochemical routes. Site directed mutagenesis of 1, 3-propanediol oxidoreductase at Asp48 residue relaxes the cofactor specificity of 1, 3-propanediol toward NADH and NADPH, thereby increasing enzyme efficiency (Ma et al., 2010). NADPH-dependent hydroxybenzoate hydroxylase from *Pseudomonas fluorescens* was also subjected to site directed mutagenesis that increases its cofactor specificity for NADH. This was the first report on the coenzyme reversion of a flavoprotein aromatic hydroxylase (Eppink et al., 1999).

### Change in the catalytic activity of the enzyme

Genetic as well as chemical technologies offer tremendous scope of modifying the basic characteristics of enzymes, e.g., activity, stability, thermal sensitivity etc., to enhance their performance and to make them more suitable for industrial as well as research applications. Aspartate aminotransferases have central role in amino acid metabolism. Out of 13 random mutations, six mutations (Table 1) contributed maximum to the increased activity. Only six amino acids of the enzyme were directly interacting with substrate and results showed that directed molecular evolution is a powerful technique for enzyme redesigning if an adequate selection system is applied (Yano et al., 1998).

Tyrosine phenol lyase of microbial origin from *C. freundii* uses pyridoxal 5′ phosphate as cofactor and catalyzes the β-elimination of L-tyrosine to produce phenol and ammonium pyruvate. The substitution of Asn185Ala results in formation of a mutant, which has decreased affinity for substrate due to destability of quinonoid structure, formed between them (Barbolina et al., 2000). Similarly, Tyr71Phe was substituted in Tyrosine phenol- lyase and as a result no detectable activity for β-elimination of L-tyrosine was observed and also decreased activity for other substrate was observed (Chen H. Y. et al., 1995). Similarly, Asp124Thr and His448Phe mutations in tryptophan indole-lyase of *Proteus vulgaris*, have little or no β-elimination activity with L-Tyr or 3-fluoro-L-Tyra substrate, but retain significant elimination activity with S-(o-nitro phenyl)-L-cysteine, S-alkyl-L-cysteines, and β-chloroalanine (Demidkina et al., 2002). In *E. coli* site-saturation mutagenesis was done and many mutants were made by substituting the active site loop residues. Out of those mutants only some of the substitution mutations increased β–galactosidase activity that is discussed in the Table 1 (Geddie and Matsumura, 2004).

## Thermodynamics of the *in vitro* design of enzyme

In continuation of above discussion, it is also important to study the thermodynamic parameters like ΔG, ΔCp, ΔH involved in the evolutionary path of these enzymes. Using thermodynamic divergence of evolutionary path of mesophilic and thermophilic Ribonuclease H1 from their common ancestor has been traced (Hart et al., 2014). Analyzing its mutants enormous amount of information about the critical positions in enzyme-substrate selectivity can be collected. This is useful in finding their evolution mechanisms i.e., how these enzymes evolved from their ancestors using ancestral sequence reconstruction etc. (Hart et al., 2014). Directed evolution technology to select an enzyme with desired characteristics is one of the earliest and successful methods such as half-life (*t_1/2_*) and activity (*k*_*cat*_) of a cold-adapted Lipase B isolated from *Candida antarctica* were significantly improved from 8 to 211 min and 84 min^−1^ to 1900 min^−1^, respectively.

## Conclusions

Combined computational and *in vitro* evolution approaches can be used for the study of evolution of novel bioactivity in the natural enzymes as well as redesigning of the function of the enzyme. DNA shuffling, site-saturation mutagenesis, and site-directed mutagenesis are used can be used to redesign the active site of an enzyme, which can alter its efficiency. Artificial alteration of enzyme can be very useful in changing the catalytic efficiency of enzymes, cofactor specificity, and substrate specificity. Many new enzymes that do not occur naturally can also be developed, which are more efficient. Using these approaches, enzymes can be engineered according to the requirement. For example, if there is defect in any enzyme, the existing related enzyme or isozymes could be engineered in such a way so that it can work in place of defected enzyme. Moreover, the evolutionary relation and thermodynamic system drifts over evolutionary time can also be studied.

## Future prospects

Engineered enzymes revolution has been brought about after the discovery of extremophiles, organisms surviving, and thriving in extreme environmental conditions like high temperature, low temperature, high salt, and pH etc. Preliminary studies with extremophile enzymes have provided the researchers vital information about the mechanisms of their adaption of the extreme environments and different catalytic activity. These studies revealed the biochemical and structural features of these enzymes, which facilitated scientists to engineer the mesophilic enzymes to attain thermo- or cryo-stability. Similarly, engineered thermophilic and psychrophilic enzymes attain substrate specificity and activity of mesophilic enzymes and vice versa. For example, thermostable hydrolases (used in starch, food, pharmaceutical, leather, and textile industries), chitinases (used in food, cosmetics, pharmaceuticals, agrochemicals related industries), and phytases (release phosphorus from the phytate are used in animal feed); and cryostable lipases, proteases, phytases, glucanases, and xylanases are used with animal feed; pectinases and xylanases are used in starch hydrolysis at low temperatures; lipases and proteases used in detergent and cleaning industry; lipases are being used in biofuel and energy producing industry etc. Similarly, research activities can also result in the development and production of numerous engineered enzymes with application in industrial processes, therapeutics and household products including food industry, biodiesel, and bioremediation.

## Author contributions

Conceived, Designed, Proofread, and corrected: VT.

### Conflict of interest statement

The author declares that the research was conducted in the absence of any commercial or financial relationships that could be construed as a potential conflict of interest.
